# Development of [^11^C]vemurafenib employing a carbon-11 carbonylative Stille coupling and preliminary evaluation in mice bearing melanoma tumor xenografts

**DOI:** 10.18632/oncotarget.16321

**Published:** 2017-03-17

**Authors:** Paul Slobbe, Albert D. Windhorst, Kevin Adamzek, Marije Bolijn, Robert C. Schuit, Daniëlle A.M. Heideman, Guus A.M.S. van Dongen, Alex J. Poot

**Affiliations:** ^1^ Department of Radiology and Nuclear Medicine, VU University Medical Center, Amsterdam, The Netherlands; ^2^ Department of Otolaryngology/Head and Neck Surgery, VU University Medical Center, Amsterdam, The Netherlands; ^3^ Department of Pathology, VU University Medical Center, Amsterdam, The Netherlands

**Keywords:** vemurafenib, BRAF, V600E, personalized medicine, PET

## Abstract

Over the last decade kinase inhibitors have witnessed tremendous growth as anti-cancer drugs. Unfortunately, despite their promising clinical successes, a large portion of patients does not benefit from these targeted therapeutics. Vemurafenib is a serine/threonine kinase inhibitor approved for the treatment of melanomas specifically expressing the BRAF^V600E^ mutation. The aim of this study was to develop vemurafenib as PET tracer to determine its potential for identification of tumors sensitive to vemurafenib treatment. Therefore, vemurafenib was labeled with carbon-11 and analyzed for its tumor targeting potential in melanoma xenografts Colo829 (BRAF^V600E^) and MeWo (BRAF^wt^) using autoradiography on tissue sections, *in vitro* tumor cell uptake studies and biodistribution studies in xenografted athymic nu/nu mice. [^11^C]vemurafenib was synthesized in 21 ± 4% yield (decay corrected, calculated from [^11^C]CO) in > 99% radiochemical purity and a specific activity of 55 ± 18 GBq/μmol. Similar binding of [^11^C]vemurafenib was shown during autoradiography and cellular uptake studies in both cell lines. Plasma metabolite analysis demonstrated > 95% intact [^11^C]vemurafenib *in vivo* at 45 minutes after injection, indicating excellent stability. Biodistribution studies confirmed the *in vitro* results, showing similar tumor-to-background ratios in both xenografts models. These preliminary results suggest that identification of BRAF^V600E^ mutations *in vivo* using PET with [^11^C]vemurafenib will be challenging.

## INTRODUCTION

The discovery of driver oncogenes in cancer has paved the way for the development of novel, selective therapies in cancer treatment. The increased understanding of the specific mutations and signaling pathways in tumor formation and proliferation has led to the development of tailor-made targeted therapeutics. One particular type of enzymes, namely kinases, have been particularly well studied over the last few decades and numerous drug discovery programs have been aimed at this class of targets [1].

Kinases comprise a large family of proteins that play an essential role in the signal transduction pathways of cells. Two subcategories can be distinguished, being the receptor kinases and the non-receptor or cytosolic kinases. The receptor kinases are transmembrane complexes which bind extracellular signaling ligands, upon which the receptor oligomerizes and the downstream signaling cascade is activated via the kinase domain. This cascade often consists of various cytosolic kinases. These are phosphorylated sequentially which leads to a cellular response, such as proliferation. A prominent example in this is the RAS-RAF-MEK-ERK pathway [2].

Kinases are heavily involved in cell signaling, growth and differentiation and therefore aberrant activation of kinases often leads to tumor formation. The development of therapeutics targeting kinases has received a lot of interest over the last two decades. An important category of targeted therapeutics comprises the small molecule kinase inhibitors which act intracellularly by competing with ATP for its binding site at the kinase domain of the receptor complex and thereby inhibit downstream signaling. Requirements for kinase inhibitors are good cell uptake and high affinity binding to the kinase domain. A drawback of targeted drugs like kinase inhibitors is a lack of selectivity (cross reactivity with healthy tissue also expressing the targeted receptors) and therefore, currently, the most effective kinase inhibitors are aimed at specific oncogenic mutations in the kinase that are not expressed in healthy tissue. This defines their selectivity over wild type kinase inhibitors. On the other hand if the mutation is not present in the tumor, the kinase inhibitor is less active and therapy is often only marginally successful, while side effects are still induced [3].

As an example, for Tyrosine Kinase Inhibitors (TKIs) targeting the Epidermal Growth Factor Receptor (EGFR), as used in the treatment of non-small cell lung cancer, the underlying reason for this inter-patient variability is best understood. Activating mutations in the kinase domain of EGFR dictate the effectiveness of the TKIs currently on the market (such as: erlotinib, gefitinib and afatinib) [4, 5]. Accordingly, molecular testing in pathology to identify the specific molecular alterations in the tumor has become a critical part of the process of selecting patients for appropriate treatments [6].

In recent publications the use of Positron Emission Tomography (PET) with radiolabeled kinase inhibitors, as a tool to study TKI disposition *in vivo* has been developed by others and us [7–9]. By labeling FDA approved kinase inhibitors with a PET-isotope in an inert manner (i.e., no structural modifications are performed), the *in vivo* biodistribution, pharmacokinetics and tumor targeting can be determined in a noninvasive manner at tracer level. The most successful examples to date include the use of [^11^C]erlotinib, which was able to distinguish between sensitizing mutations of EGFR and wild type EGFR in non-small cell lung cancer patients, and [^18^F]afatinib showing a promising similar preclinical targeting profile in tumor bearing mice [10–14]. The aim of this study was to extend this concept to vemurafenib, a serine/threonine kinase inhibitor. To our knowledge this report is the first time a mutated serine/threonine kinase was targeted for imaging with an FDA approved drug.

**Figure 1:**
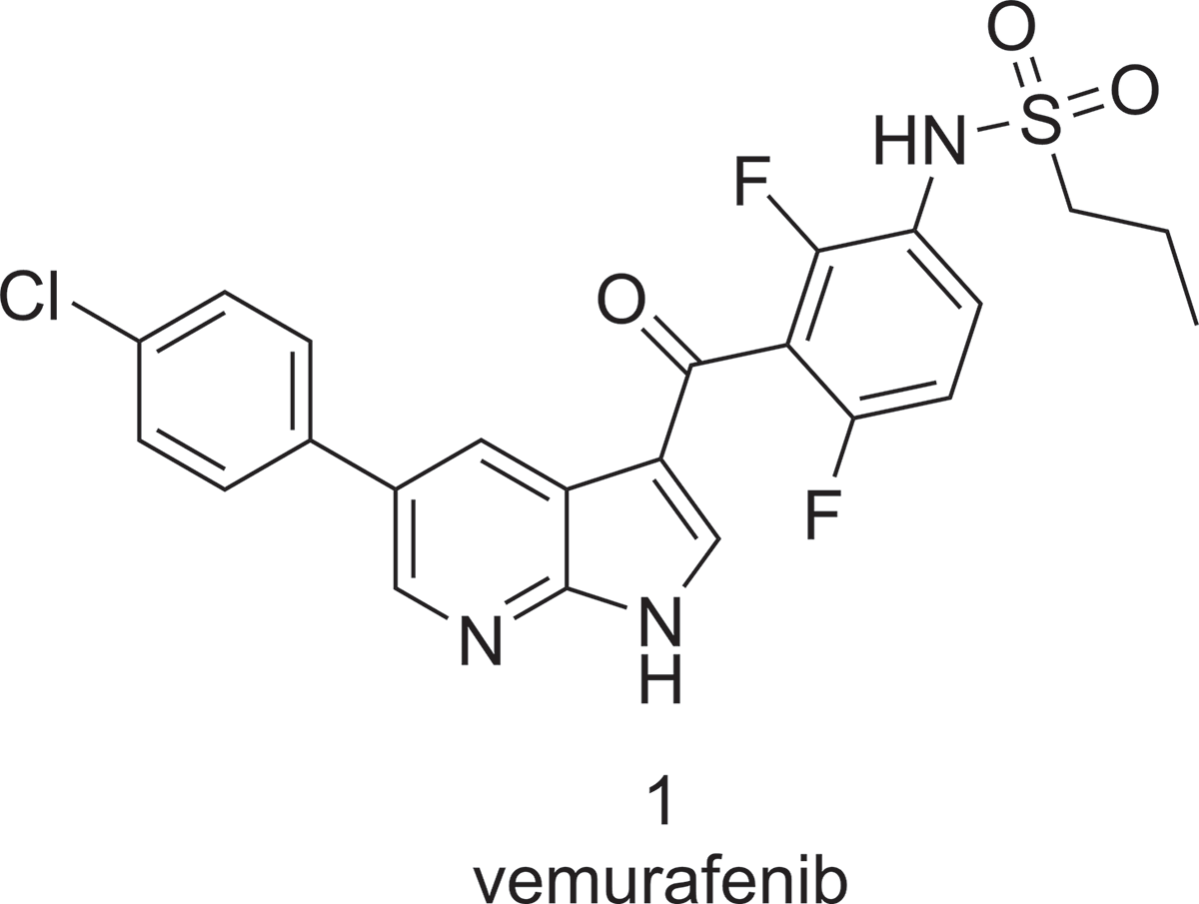
Chemical structure of vemurafenib

Vemurafenib (1, Zelboraf, Roche, Figure 1) is a mutation selective serine/threonine kinase inhibitor developed to specifically inhibit mutated BRAF in the RAS-RAF-MEK-ERK pathway. The V600E mutation of *BRAF* (in which a valine is substituted for a glutamic acid at codon 600) was discovered as an oncogenic driver mutation in 2002 when this mutation was observed in different cancers. This mutation occurs in the activation loop of BRAF and substantially increases kinase activity to drive the proliferation of cancer cells. Expression of mutated BRAF is described for approximately 50% of all melanomas and is also observed in varying prevalence in other types of cancers, e.g. colorectal cancer, non-small cell lung cancer and gastric cancer [15]. Vemurafenib demonstrated good efficacy in various melanoma and colorectal xenografts *in vivo*, which are BRAF^V600E^ positive [16]. Clinical development of vemurafenib started in 2006 and in a phase I trial with 32 patients an unprecedented 81% unconfirmed overall response rate was observed, leading to the pivotal phase III trial that showed a significant improvement in the duration of survival in patients who received vemurafenib *vs* patients treated with dacarbazine, with a 63% reduction in the risk of death [15]. Vemurafenib was approved for treatment of late stage melanoma in 2011. Despite the successes of vemurafenib, patient selection in the case of therapy is of the utmost importance, as in tumors expressing wild type BRAF it has an inverse mode of action. In those cases experimental evidence suggests that vemurafenib actually leads to increased tumor cell proliferation. Accordingly, effective molecular testing for BRAF is in place in pathology laboratories.

Halaban *et al*. demonstrated this dual action mode of vemurafenib in BRAF^WT^ cell cultures isolated from primary or metastatic melanoma tumor tissue [17]. In BRAF^V600E^ cells, treatment with vemurafenib abolished ERK1/2 phosphorylation leading to inhibition of cell growth. Treatment of BRAF^wt^ cells with vemurafenib, however, lead to rapid phosphorylation of ERK1/2 and MEK [17]. These results were confirmed in additional studies using established tumor cell lines (also used in this study) and this was shown also to be the case for structurally unrelated inhibitors targeting BRAF^V600E^, thereby demonstrating this effect not to be specific for vemurafenib only [18]. It has been hypothesized that RAS-GTP dependent activation of RAF1 (or CRAF) occurs in these cells, likely by heterodimerization with BRAF, resulting in activation of the MEK-ERK signaling cascade and ultimately leading to proliferation.

The aim of this study was to develop [^11^C]vemurafenib as a PET imaging agent for the identification of BRAF^V600E^ expressing tumors. To this end, a radiolabeling route was developed and preclinical studies with tumor cells and tissues performed. Finally, the *in vivo* targeting potential of [^11^C]vemurafenib was evaluated in both wild-type and mutant xenografts as a prelude to the use [^11^C]vemurafenib in clinical PET for patient stratification.

## RESULTS AND DISCUSSION

### Synthesis of [^11^C]vemurafenib

**Scheme 1:**
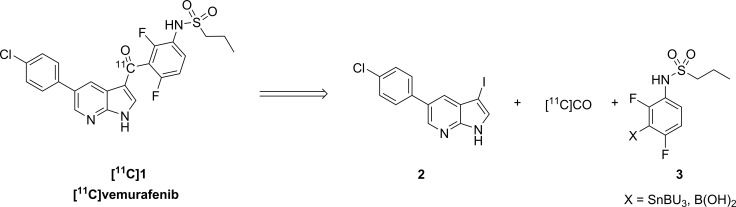
Retrosynthetic analysis of [^11^C]vemurafenib via palladium catalyzed carbonylative cross coupling reactions

Vemurafenib (1) can in theory be labeled with a PET-isotope on several positions. For labeling with carbon-11 the carbonyl position is amenable to radiolabeling via a carbonylative cross coupling reaction (Scheme 1). Many palladium cross coupling reactions are suitable for combination with carbon-monoxide insertions (e.g. Heck, Negishi, Sonogashira and others) [19], however, development of these chemical transformations for radiochemistry is still in its early stages. For the synthesis of [^11^C]vemurafenib two types of palladium mediated cross coupling reactions with [^11^C]CO-insertion were selected as potential synthesis routes, being the Suzuki and the Stille coupling [20, 21]. Both provide the motif required and examples of radiochemical variants of these reactions have appeared in literature [21]. A major challenge in [^11^C]carbon monoxide insertion is the substoichiometric amount of [^11^C]CO present as opposed to the pressurized CO atmosphere in which these reactions are traditionally performed. The retrosynthesis for both routes is depicted in Scheme 1 and involves the cross coupling of an aryl halide (such as 2) with an aryl boronic acid (Suzuki coupling) or tributylstannane (Stille coupling) as depicted in structure 3. As aryl halide the aryl iodide was selected, since it is considered to be the most reactive halide with regard to oxidative addition to palladium [22], being the first step in the catalytic cycle. With respect to 3 the tributyl stannane was chosen as this compound could be readily synthesized whereas the boronic acid for the Suzuki coupling has been proven to give problems with respect to isolation in sufficient purity. Therefore, the Stille coupling with [^11^C]CO insertion was chosen as synthesis route for [^11^C]vemurafenib. To our knowledge only few reports exist in which these types of radiochemical reactions are described and generally high concentrations of precursors are employed to achieve good radiochemical yields [23, 24]. Finally, literature data suggests that the rate-limiting step in this reaction is the transmetalation of the organostannane with palladium [25, 26].

As an alternative for [^11^C]vemurafenib, fluorine-18 labeling of vemurafenib was also considered. The fluorine atoms, however, are in a challenging position to label due to the fact that these are relatively inactivated. Nevertheless, radiofluorination of similar moieties has been reported [27]. Additionally, novel methodology to fluorinate these inactivated positions are being reported and such methods could prove effective in the synthesis of a fluorine-18 derivative of vemurafenib [28].

### Precursor synthesis

**Scheme 2:**
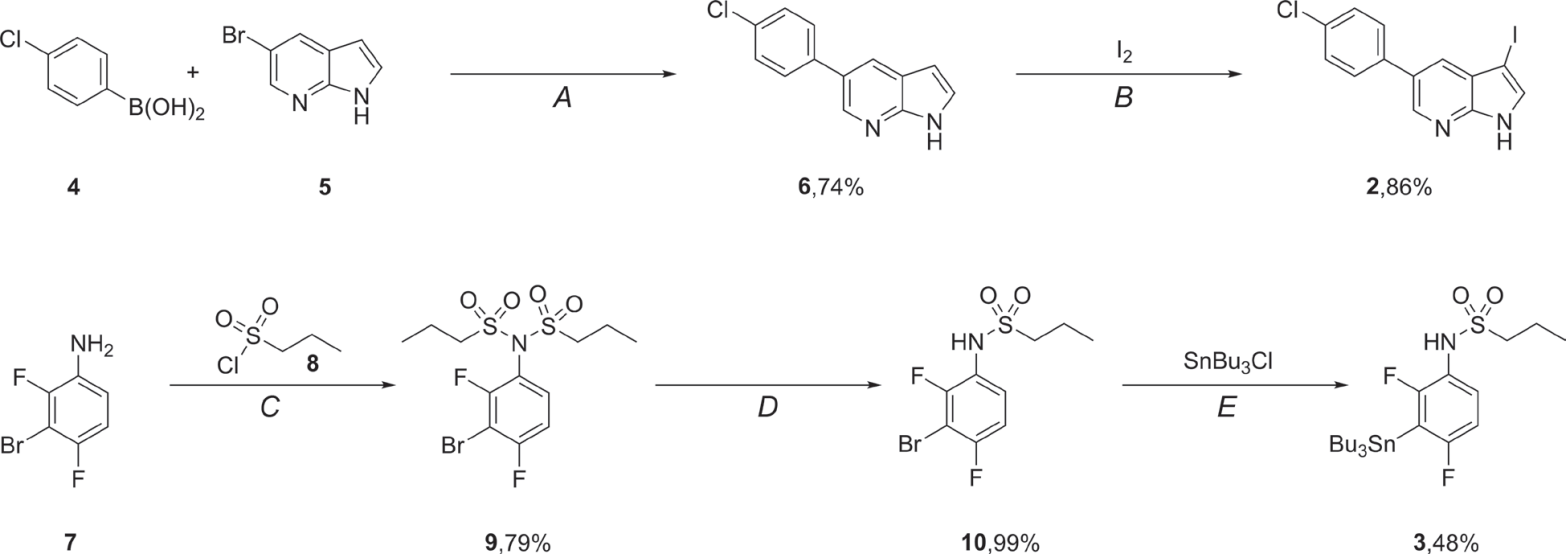
Synthesis of precursors required for [^11^C]vemurafenib Reagents and conditions: (**A**) Pd(dppf)Cl_2_·CH_2_Cl_2_, K_2_CO_3_, dioxane/H_2_O, 80°C, 16 h; (**B**) DMF, 20°C, 2 h; (**C**) Et_3_N, CH_2_Cl_2_, 20°C, 16 h; (**D**) NaOH, MeOH/H_2_O, 20°C, 2 h; (**E**) *n*-BuLi, THF, −78°C-rt, 16 h.

First the two precursors namely, aryliodide 2 and stannane 3 needed to be synthesized (Scheme 2) prior to the Stille coupling with [^11^C]CO. The synthesis of aryliodide 2 starts with a Suzuki reaction to couple *para*-chlorophenylboronic acid (4) to 5-bromoazaindole (5) to yield the cross coupled product 6. 6 was subjected to iodination with elemental iodine to obtain the desired aryl iodide 2 in a satisfying yield of 64% over 2 steps.

The synthesis of the second precursor, 3, starts with double alkylation of 3-bromo-2,4-difluoro-aniline 7 with propane-1-sulfonylchloride (8) affording 9 in 79% yield. One of the introduced propanesulfonyl side chains is subsequently removed under basic conditions to furnish mono-substituted aniline 10 in 99% yield. Introduction of the tributylstannane moiety is achieved via a base promoted halogen lithium exchange and subsequent substitution with tributyltinchloride in 48% yield to provide the second precursor (3) required for radiolabeling.

### Radiochemistry

[^11^C]CO was produced by reduction of cyclotron produced [^11^C]CO_2_ over molybdenum at 850°C and the obtained [^11^C]CO (4686 ± 2093 MBq, *N = 15*) was subsequently transferred to a closed reaction vial containing THF, employing xenon as transfer gas by the recently developed method of Eriksson *et al*. [29]. The carbonylation reactions were limited to 5 minutes because of the limited half-life of carbon-11 of 20 minutes.

Our investigation started with the selection of a suitable combination of palladium source and co-ligand which was effective for the synthesis of [^11^C]vemurafenib (Table 1). After screening a subset of palladium source/ligand combinations the combination of Pd_2_(dba)_3_ with triphenylarsine demonstrated promising conversion radiochemical of [^11^C]CO, as assessed by analytical HPLC (Table 1, entry 4). Therefore this combination of palladium source and ligand was used for further optimization.

**Table 1:** Selection of palladium/ligand combinations screened for the synthesis of [^11^C]vemurafenib

#	Pd-source	Ligand	Solvent	Conversion (%)^a^
1	PdCl_2_(Dppf)·CH_2_Cl_2_	-	THF	< 1
2	Pd_2_(dba)_3_	Tri-o-tolylphosphine	DMSO^b^	10
3	Pd_2_(dba)_3_	Tri-o-tolylphosphine	THF	13
4	Pd_2_(dba)_3_	Triphenylarsine	THF	16

Reactions performed at 10 μmol of reagents and 1 μmol of catalyst/ligand and at 100°C for 5 minutes. ^a^ conversion determined from a sample withdrawn from the reaction mixture and analysis by analytical HPLC. ^b^ Solubility of xenon gas in DMSO is poor and therefore transfer of [^11^C]CO is limited [29].

First the ratio between Pd_2_(dba)_3_ and triphenylarsine was investigated and a substantial increase was observed when 2 equivalents of ligand were used in comparison with palladium, however, no further increase in conversion was observed at a higher ratio (Table 2, entries 1-3). The ratio between precursors aryliodide 2 and stannane 3 was set at 1:3 in favor of the stannane, to promote the transmetalation step. A substantial increase in conversion was observed when the concentration of these reagents were increased 3-fold (Table 2, entry 4), which is in good accordance with data from literature [30]. Further increase of the precursor concentrations of 2 and 3 led to precipitation and solubility issues when preparing the reaction mixture. Additional variation of the stoichiometry did not result in higher conversion towards [^11^C]vemurafenib (Table 2, entries 5-8). The 3-fold excess of stannane 3 indicates that the rate limiting step in this Stille coupling is indeed the transmetalation, as significantly higher conversions were obtained than when equimolar amounts of both reagents were used or when an excess of aryl iodide was used (compare Table 2, entry 4 with 7 and 8), which is in accordance with data reported on Stille reactions [25, 26]. After HPLC purification and formulation of [^11^C]vemurafenib, these optimized reaction conditions provided an isolated yield of 21 ± 4% (corrected for decay, calculated from [^11^C]CO) in > 99% radiochemical purity, sufficient specific activity of 55 ± 18 GBq/μmol in 35 ± 2 minutes of synthesis time as an i.v. injectable solution.

**Table 2:** Optimization of stoichiometry

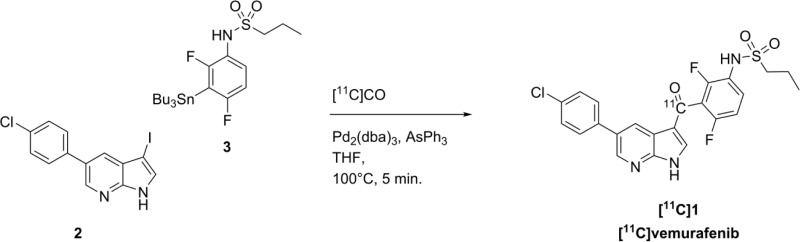							
#	2 (μmol)	3 (μmol)	Ratio	Pd_2_(dba)_3_ (μmol)	AsPh_3_ (μmol)	Ratio	Conversion (%)^a^
1	10	30	3	10	10	1	7
2	10	30	3	10	20	2	17
3	10	30	3	10	30	3	18
**4**	**33**	**99**	**3.0**	**10**	**20**	**2**	**41**
5	30	60	2.0	10	20	2	35
6	56	70	1.3	10	20	2	23
7	70	70	1.0	10	20	2	27
8	60	45	0.75	10	20	2	14

^a^Conversion determined by first purging of excess [^11^C]CO from the reaction vial and subsequently a sample was withdrawn from the reaction mixture and analyzed by analytical HPLC. Expressed as average of two independent experiments.

### Cell lines for evaluation of [^11^C]vemurafenib binding *in vitro* and *in vivo*

**Figure 2:**
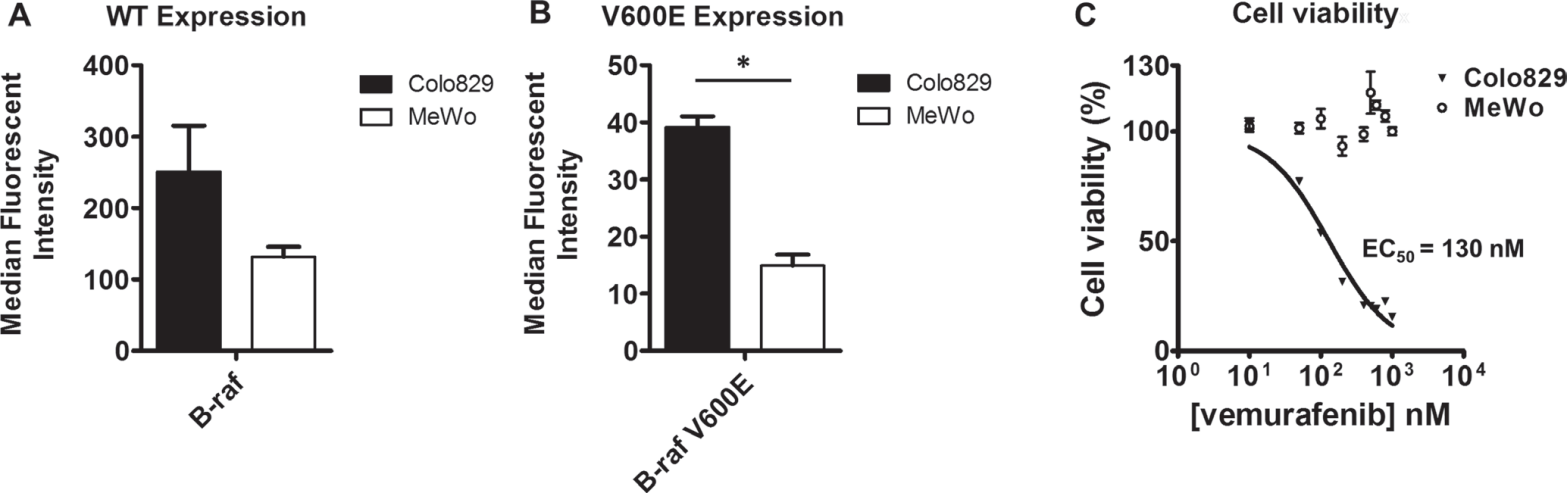
(**A**) FACS analysis for BRAF expression using a BRAF antibody that recognizes both mutated as well as wild type BRAF; (**B**) FACS analysis for BRAF expression using a BRAF antibody that recognizes V600E mutated BRAF; (**C**) Representative cell viability curve after vemurafenib treatment *in vitro* of MeWo and Colo829 cells. Median fluorescent intensity was compared between the cell lines by using a two-tailed Student's *t-test* (* = statistically significant; *p* < 0.05).

Having a reliable synthesis route to obtain [^11^C]vemurafenib, cell lines had to be selected to evaluate the tumor targeting potential of [^11^C]vemurafenib. Two melanoma cell lines were selected based on reported mutational status of BRAF: Colo829 (BRAF^V600E^) and MeWo (BRAF^wt^). Both cell lines were analyzed by Fluorescence Activated Cell Sorting (FACS) using a BRAF antibody (recognizing both wild-type and BRAF^V600E^) and a BRAF^V600E^ selective antibody for assessment of target expression (Figure 2A/2B) [16, 31]. This demonstrated that the BRAF^wt^ antibody detects expression in both cell lines, with a higher expression for Colo829 when compared to MeWo, although this was not statistically significant (Figure 2A). The BRAF^V600E^ antibody however, detects expression in the Colo829 cell lines, which is significantly higher when compared to the MeWo cells (Figure 2B). Furthermore, both the cells as well as the xenografts derived from the cell lines were sequenced to confirm the expression of BRAF and the mutational status thereof. This revealed that Colo829 cells and xenografts contained *BRAF* mutation c.1799T > A; p. (V600E) and MeWo cells and xenografts were wild-type. FACS analysis on the other hand with mutation specific antibodies demonstrated expression of both isoforms in either cell line, indicating that there is a heterogeneous expression of both wild-type and mutated BRAF. This was an unexpected result since there was a clear difference in sensitivity between the cell lines towards vemurafenib treatment as observed in cell viability assays (*vide supra)*, which made us pursue [^11^C]vemurafenib PET as potential predictive value. We verified the sensitivity of the cell lines towards vemurafenib treatment using-titer blue assays. The BRAF^V600E^ expressing cell line (Colo829) was found to be sensitive to vemurafenib treatment (IC_50_ = 130 nM), while the BRAF^wt^ expressing cell line (MeWo) was not, (no IC_50_ was observed, Figure 2C). So while the BRAF^V600E^ kinase is sensitive to vemurafenib treatment, it appears that a certain level of mutated protein expression is required for this sensitivity, as both cell lines expressed the mutation to an extent, as determined by FACS. In contrast sequencing only detected the V600E mutation in the Colo829, indicative of higher expression. Taken together the complex nature of the cell lines warrants careful interpretation of the results. The next step was to determine if the sensitivity observed could be predicted by [^11^C]vemurafenib binding and biodistribution, regardless of the complex biology.

### *In vitro* evaluation of [^11^C]vemurafenib

**Figure 3:**
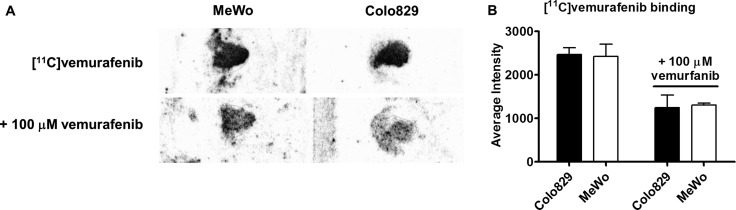
(**A**) Representative autoradiography images of tumor sections of MeWo and Colo829 xenograft tumor sections incubated with [^11^C]vemurafenib (top) and with [**^11^**C]vemurafenib co-incubated with vemurafenib (100 μM); (**B**) Quantification of blocking experiment. Binding was compared between the cell lines by using a two-tailed Student's *t-test* and found not to be statistically significant in both the blocked or unblocked conditions.

To determine the binding potential of [^11^C]vemurafenib to tumor tissue expressing BRAF^wt^ or BRAF^V600E^, autoradiography studies on xenograft sections and *in vitro* cellular uptake studies were performed. Sections of tumor xenografts derived from the aforementioned cell lines were incubated with [^11^C]vemurafenib. This demonstrated no statistically significant difference in binding to both cell types (Figure 3). Upon co-incubation with isotopically unmodified vemurafenib (100 μM), binding decreased significantly although significant non-specific binding remained (Figure 3B). MeWo demonstrated 65.1 ± 3.1% binding when compared to unblocked conditions and Colo829 showed 51.2 ± 11.2% (Figure 3B). These results suggest similar binding of [^11^C]vemurafenib to tissue expressing BRAF^wt^ and BRAF^V600E^.

**Figure 4:**
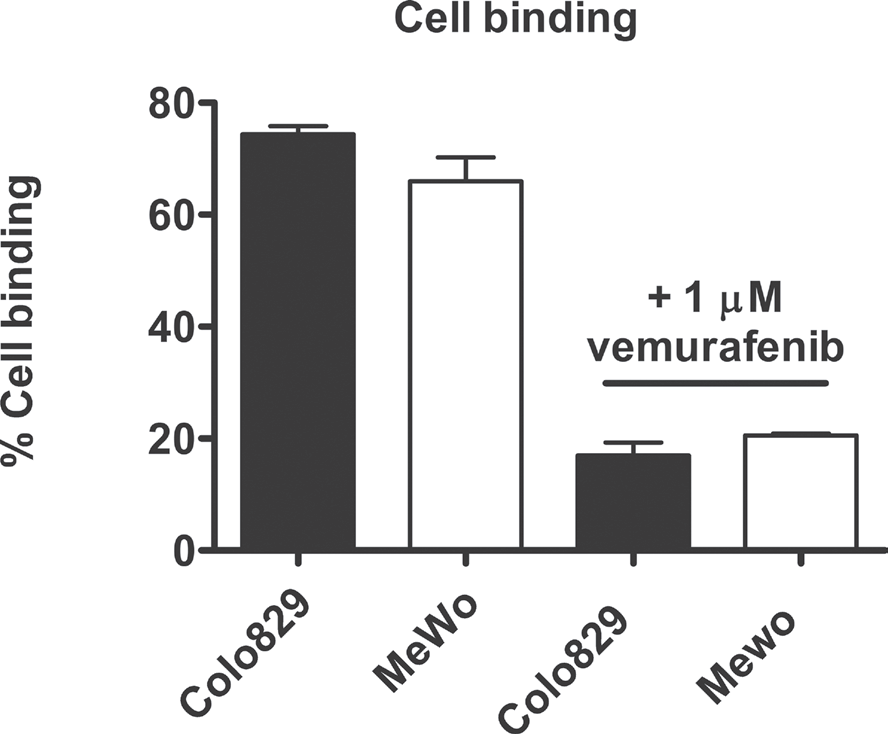
Quantification of cell bound + internalized fraction of [^11^C]vemurafenib after incubation with tumor cells Binding was compared between the cell lines by using a two-tailed Student's *t*-test and found not to be statistically significant in both the blocked or unblocked conditions.

To determine binding of [^11^C]vemurafenib *in vitro* to intact tumor cells a cell uptake study was performed by incubation of tumor cells in suspension with [^11^C]vemurafenib, followed by removal of the supernatant and washing of the cells with PBS (phosphate buffered saline). High binding was observed in both cell lines, with Colo829 cells demonstrating slightly higher binding when compared to MeWo cells (Figure 4, 74.3 ± 1.5% vs 61.6 ± 8.2%), however, this was not statistically significant. Upon pretreatment of the cells with isotopically unmodified vemurafenib a large decrease was observed in cell binding (to 17.0 ± 2.3 for Colo829 and 20.6 ± 0.4% for MeWo cells), however, also in this case no statistically significant difference was found between the two cell lines. These experiments demonstrated that [^11^C]vemurafenib is able to bind to tumor tissue and is taken up by tumor cells, however, both autoradiography and cellular uptake studies demonstrated similar binding of [^11^C]vemurafenib to both cell types, prompting further investigation using [^11^C]vemurafenib in biodistribution studies with xenograft bearing mice. While *in vitro* experiments can be of great value to predict tracer target binding, the experiments exclude many of the factors observed *in vivo*, for PET-tracer validation. Therefore *in vivo* experiments are of the utmost importance for PET-tracer validation. Furthermore, in the *in vitro* setting the tumor tissue and cells are constantly exposed to the tracer during incubation, which is not the case in an *in vivo* setting where clearance of the tracer or uptake in non-target tissue influences and lowers the tumor exposure to the tracer. It might well be that the difference in binding of vemurafenib between the cell lines can only revealed *in vivo*, due to the multitude of pharmacokinetic factors that influence tracer distribution such as the lack of constant exposure of the tissue to the tracer, which is the case in *in vitro* experiments. Next to that, the competition for endogenous ligands (such as ATP) and other factors (e.g. metabolism) are better represented in an *in vivo* model, which also influences tracer binding. Therefore exploratory *in vivo* experiments are warranted to determine the potential of [^11^C]vemurafenib as PET tracer.

### *In vivo* stability

To determine the *in vivo* stability of [^11^C]vemurafenib, before the start of *in vivo* experiments, a plasma metabolite analysis was performed in non-tumor bearing athymic nu/nu mice. The mice were injected i.v. (via ocular plexus) with 20–25 MBq of [^11^C]vemurafenib (corresponding to 0.36–0.45 nmol of vemurafenib) and sacrificed at 15 and 45 minutes post injection followed by blood sample collection. The non-polar and polar fractions were separated using solid phase extraction. Analysis of the blood plasma (Table 3) revealed excellent stability of [^11^C]vemurafenib with > 95% stability 45 minutes after injection. HPLC analysis of the non-polar fraction confirmed the absence of metabolites.

**Table 3:** Plasma metabolite analysis after injection of [^11^C]vemurafenib in non-tumor bearing mice

Time (p.i.)	Polar metabolites (%)	Parent (%)
15	2.2 ± 1.9	97.8 ± 1.9
45	4.1 ± 4.4	95.9 ± 4.4

No non-polar metabolites were observed upon HPLC analysis of the non-polar fraction, which was confirmed by off-line counting of HPLC fractions. Recovery of radioactivity was > 95%.

### *Ex vivo* biodistribution studies

**Figure 5:**
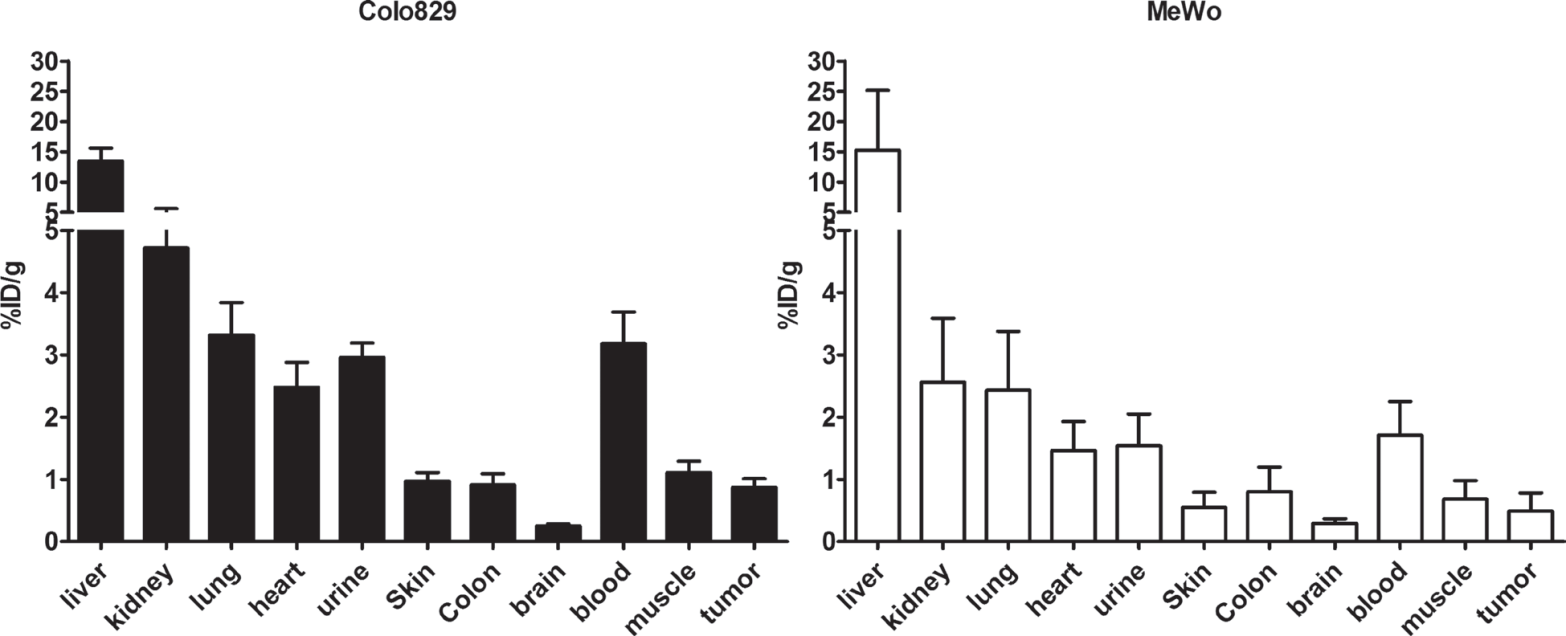
Biodistribution 60 minutes post injection of [^11^C]vemurafenib in Colo829 (left, BRAF^V600E^) and MeWo (right, BRAF^wt^) xenograft bearing mice (*n* = 3 per time point) after administration of 20–25 MBq (corresponding to 0.36–0.45 nmol of vemurafenib) under isoflurane anesthesia Tumor uptake (%ID/g) was compared between the cell lines by using a two-tailed Student's *t*-test and found not to be statistically significantly different.

To evaluate the *in vivo* tumor targeting potential an *ex vivo* biodistribution study was performed in mice bearing the aforementioned melanoma xenografts. Tumor bearing mice were injected with 20–25 MBq of [^11^C]vemurafenib (corresponding to 0.36–0.45 nmol of vemurafenib) and sacrificed 60 minutes post injection. Subsequently organs of interest were excised and counted for activity. [^11^C]vemurafenib accumulation in the different organs and the tumor are depicted in Figure 5 and expressed as injected dose per gram of tissue (%ID/g).

High liver uptake was observed for [^11^C]vemurafenib, which is more frequently observed for small molecules due to their catabolism in the liver. Uptake in the kidney and urine was observed, indicating renal excretion is a clearance route of [^11^C]vemurafenib. Highly perfused organs such as heart and lungs also demonstrated high uptake, similar to blood levels. Interestingly, the biodistribution also demonstrated relatively high blood levels of [^11^C]vemurafenib after 60 minutes (2–3%ID/g), which is usually lower for small molecules due to rapid blood clearance of this type of PET-tracer. Tumor uptake was modest for both xenograft lines investigated with an uptake of 0.49 ± 0.29%ID/g in MeWo tumors and 0.87 ± 0.14 ID/g in Colo829 tumors. While in an absolute sense the sensitive BRAF^V600E^ xenografts demonstrated slightly higher uptake, this was not statistically significant and a comparison of the resulting tumor-to-background ratios clearly demonstrated similar uptake ratios across both xenograft lines (Table 4). The relevant surrounding normal tissues for melanomas being muscle, blood and skin were nearly identical when tumor-to-tissue ratios were calculated for [^11^C]vemurafenib and in all cases the ratio never exceeded 1. The obtained *in vivo* results, however, are in good accordance with the results observed during the *in vitro* studies, in which also similar uptake was observed in both studied tumor types. This preliminary data indicates that [^11^C]vemurafenib might be unsuitable for the *in vivo* detection of BRAF^V600E^ tumors.

**Table 4:** Tumor-to-normal tissue ratios at 60 min p.i

	Colo829 (BRAF^V600E^)	MeWo (BRAF^WT^)
**Tumor-to-muscle**	0.78 ± 0.18	0.72 ± 0.21
**Tumor-to-blood**	0.27 ± 0.06	0.29 ± 0.19
**Tumor-to-skin**	0.90 ± 0.19	0.89 ± 0.28

The low tumor uptake and undesired low tumor-to-normal tissue ratios might be explained by the fact that [^11^C]vemurafenib is unable to reach its target in the tracer amount administered during biodistribution studies. Therapeutic dosing of vemurafenib is done in the order of 50 mg/kg (corresponding to circa 2.55 μmol of vemurafenib) repeatedly in preclinical efficacy studies leading to constant higher plasma concentrations and subsequently higher tumor exposure. Furthermore, prior to target binding, [^11^C]vemurafenib first has to cross the cellular membrane and compete with high intracellular ATP concentrations [32], further hampering target engagement. It might well be that due to the fact that BRAF is a cytosolic protein target, engagement of the tracer to the target is more challenging than for example with a transmembrane receptor such as EGFR. Finally, it was recently reported that vemurafenib is a substrate for ABC-cassette efflux transporters (e.g. P-gp and BCRP) promoting efflux of xenobiotics from the tumors and thereby contributing to multidrug resistance [33, 34]. The trace amounts of [^11^C]vemurafenib could be readily transported out of the cell in this manner, thereby explaining the low tumor uptake and the relatively high blood values observed in biodistribution studies. Finally as described in the introduction, paradoxical activation of the RAS-RAF-MEK-ERK is observed in BRAF^wt^ cell lines after vemurafenib treatment indicating that uptake in these tumor cells can also be expected providing a possible explanation for the similar tumor-to-normal tissue ratios observed in both BRAF^wt^ and BRAF^V600E^ xenografts. Further preclinical experiments are needed, however, to determine the applicability of [^11^C]vermurafenib as PET-tracer. These experiments might include additional cell lines (well characterized), blocking studies *in vivo* with unlabeled vemurafenib and biodistribution studies in the presence of MDR pump blocking agents.

We have reported a reliable synthesis to obtain [^11^C]vemurafenib as PET tracer using a [^11^C]CO carbonylative Stille coupling. *In vitro* autoradiography on xenograft sections and cell uptake experiments with BRAF^V600E^ Colo829 cells and BRAF^wt^ MeWo cells demonstrated similar binding to the BRAF mutated tumor tissue when compared to wild-type. *In vivo* plasma stability studies demonstrated excellent metabolic stability of [^11^C]vemurafenib, which is therefore ideally suited for *in vivo* experiments. *Ex vivo* biodistribution studies demonstrated modest uptake of [^11^C]vemurafenib, not discriminating the BRAF^V600E^ mutation. Altogether, the preliminary data presented herein indicate that PET with [^11^C]vemurafenib is challenging as a tool to identify tumors harboring the BRAF^V600E^ mutation. Additional preclinical experiments are required using a panel of better characterized cell lines with respect to BRAF expression (for example genetically less heterogeneous) and [^11^C]vemurafenib at different doses to determine the full potential of the tracer. This report describes the first step towards determining the potential of employing an FDA approved pharmaceutical targeting a mutated BRAF variant as a TKI-PET tracer.

## MATERIALS AND METHODS

### General

All reactions were carried out under atmospheric conditions unless otherwise stated and all reagents and solvents were supplied by Sigma-Aldrich (St. Louis, USA) and Biosolve BV (Valkenswaard, the Netherlands) and used as received unless stated otherwise. Reference vemurafenib was supplied by LC Labs (Woburn, Massachusetts, USA). Dimethylformamide (DMF, as received) and Tetrahydrofurane (THF, after distillation from LiAlH_4_) for anhydrous purposes were stored in dry septum capped flasks charged with molecular sieves. Thin Layer Chomatography (TLC) was performed on Merck (Darmstadt, Germany) precoated silica gel 60 F254 plates. Spots were visualized by UV quenching or ninhydrin staining. Column chromatography was carried out either manually by using silica gel 60 Å (Sigma-Aldrich) or on a Büchi (Flawil, Switzerland) sepacore system (comprising of a C-620 control unit, a C-660 fraction collector, two C601 gradient pumps and a C640 UV detector) equipped with Büchi sepacore prepacked flash columns. ^1^H and ^13^C nuclear magnetic resonance (NMR) spectra were recorded on a Bruker (Billerica, USA) Avance 500 (500.23 MHz and 125.78 MHz, respectively) with chemical shifts (δ) reported in ppm relative to the solvent. Electrospray ionization mass spectrometry (ESI-MS) was carried out using a Bruker microTOF-Q instrument in positive ion mode (capillary potential of 4500 V). Analytical HPLC was performed on a Jasco PU-2089 pump (Easton, USA) equipped with a Grace (Columbia, USA) C18 Gracesmart column (5 μm, 250 mm × 4.6 mm) and MeCN/H_2_O/TFA (65:35:0.1, *v/v/v*) as eluent at a flow rate of 1 mL·min^− 1^, with a Jasco UV-2075 UV detector (λ = 254 nm) and a NaI radioactivity detector (Raytest, Straubenhardt, Germany). Chromatograms were acquired using GINA star software (version 5.1, Raytest). Semi-preparative HPLC was carried out on a Jasco PU-2089 pump equipped with a C18 Alltima column (Grace, 5 μm, 250 mm × 10 mm) using MeCN/H2O/TFA (65:35:0.1, *v/v/v*) as eluent at a flow rate of 4 mL·min^− 1^, a Jasco UV1575 UV detector (λ = 254 nm) and a custom-made radioactivity detector. Chromatograms were acquired using ChromNAV software (version 1.14.01, Jasco). Athymic nu/nu mice were obtained from Harlan Netherlands B.V. (Horst, the Netherlands). All animal experiments were performed according to Dutch national law (‘Wet op de proefdieren’, Stb 1985, 336) and approved by the local ethics committee.

### Chemistry

#### 5-(4-chlorophenyl)-1H-pyrrolo[2,3-b]pyridine (6)

To a solution of (4-chlorophenyl)boronic acid (563 mg, 3.60 mmol) and 5-bromo-1H-pyrrolo[2,3-b]pyridine (591 mg, 3.00 mmol) in dioxane (8 mL) was added a solution of K_2_CO_3_ (498 mg, 3.60 mmol) and the mixture was stirred for 30 min. followed by the addition of Pd(dppf)Cl_2_ · CH_2_Cl_2_ (220 mg, 0.30 mmol). The mixture was stirred at 80°C overnight, upon which the volatiles were removed *in vacuo*. The obtained solid is suspended in EtOAc and washed with water. The organic layer is separated, dried (Na_2_SO_4_) and evaporated to afford the crude product, which was purified by column chromatography (Hexane/EtOAc, 6:4, v/v) to afford the product as a tan solid (509 mg, 2.23 mmol, 74%). ^1^H-NMR (500.23 Mhz, DMSO-D_6_) δ: 11.7 (br s, 1H), 8.51 (d, ^4^J_H,H_ = 1.9 Hz, 1H), 8.21 (d, ^4^J_H,H_ = 1.9 Hz, 1H), 7.52 (m, 3H), 7.74 (m, 2H), 6.51 (dd, ^3^J_H,H_ = 3.5 Hz, ^4^J_H,H_ = 1.9 Hz, 1 H). ^13^C-NMR (125.78 Mhz, DMSO-D_6_) δ: 148.6 (C_q_), 141.8 (CH), 138.4 (C_q_), 132.2 (C_q_), 129.0 (2× CH), 128.9 (2× CH), 127.6 (CH), 127.3 (C_q_), 126.7 (CH), 119.7 (C_q_), 100.2 (CH). HR-MS (ESI, 4500V): m/z calculated for C_13_H_9_ClN_4_: 228.0454, found: (M+H^+^): 229.0518.

### 5-(4-chlorophenyl)-3-iodo-1H-pyrrolo[2,3-b]pyridine (7)

In an evacuated flask, under argon atmosphere 5-(4-chlorophenyl)-*1H*-pyrrolo[2,3-b]pyridine (6, 850 mg, 3.72 mmol) and potassium hydroxide (667 mg, 11.9 mmol) were dissolved in DMF (10 mL). To this mixture was added a solution of elemental iodine (1.13 mg, 4.46 mmol) in DMF (6.5 mL). The resulting reaction mixture was stirred for 2 h at room temperature upon which the mixture was poured into ice water and the resulting precipitate was collected by vacuum filtration. The crude product was purified by flash column chromatography (Hexane/EtOAc, 1:1, v/v) to afford the title compound as a tan solid (1.13 g, 3.20 mmol, 86% yield. ^1^H-NMR (500.23 Mhz, DMSO-D_6_) δ: 12.25 (br s, 1H), 8.57 (d, ^4^J_H,H_ = 1.9 Hz, 1H), 7.88 (d, ^4^J_H,H_ = 1.9 Hz, 1H), 7.80 (m, 3H), 7.55 (d, ^3^J_H,H_ = 8.5 Hz, 2H). ^13^C-NMR (125.78 Mhz, DMSO-D_6_) δ: 148.2 (C_q_), 143.1 (CH), 137.8 (C_q_), 132.6 (C_q_), 132.1 (CH), 129.4 (2× CH), 129.3 (2x CH), 128.4 (C_q_), 126.4 (CH), 122.5 (C_q_), 55.3 (C_q_). HR-MS (ESI, 4500V): m/z calculated for C_13_H_9_ClIN_4_: 353.9421, found: (M+H^+^): 354.9465.

### N-(3-bromo-2,4-difluorophenyl)-N-(propylsulfonyl)propane-1-sulfonamide (9)

To a solution of 3-bromo-2,4-difluoroaniline (416 mg, 2 mmol) and triethylamine (585 μl, 4.20 mmol) in CH_2_Cl_2_ (10 mL) was added propane-1-sulfonylchloride (472 μL, 4.20 mmol) slowly. The mixture was stirred at room temperature under a nitrogen atmosphere overnight. The mixture was evaporated *in vacuo* and taken up in EtOAc. The organic solution was washed with KHSO_4_ (1 M, aq), NaHCO_3_ (sat, aq), brine, dried (Na_2_SO_4_) and evaporated to dryness to afford the crude product. The product was purified by flash column chromatography (Hexane/EtOAc, 9:1, v/v) to afford the title compound as a brown solid (664 mg, 1.58 mmol, 79%). ^1^H-NMR (500.23 Mhz, CDCl_3_) δ: 7.36 (m, 1H), 7.05 (ddd, ^3^J_H,F_ = 9.0 Hz, ^3^J_H,H_ = 7.4 Hz, ^4^J_H,F_ = 1.9 Hz, 1H), 3.49 (m, 2H), 1.96 (m, 4H), 1.10 (t, ^3^J_H,H_ = 7.4 Hz, 6H). ^13^C-NMR (125.78 Mhz, CDCl_3_) δ: 162.03 (C_q_), 160.0 (C_q_), 157.0 (C_q_), 132.2 (CH), 119.0 (C_q_), 112.1 (CH), 99.5 (C_q_), 57.8 (2× CH_2_), 16.9 (2× CH_2_), 12.9 (2x CH_3_). HR-MS (ESI, 4500V): m/z calculated for C_12_H_16_BrF_2_NO_4_S_2_: 418.9672, found: (M+Na^+^): 441.9478.

### N-(3-bromo-2,4-difluorophenyl)propane-1-sulfonamide (10)

*N*-(3-bromo-2,4-difluorophenyl)-*N*-(propylsulfonyl) propane-1-sulfonamide (9, 420 mg, 1 mmol) was dissolved in NaOH (2 M, aq, 5 mL) and MeOH (15 mL) and stirred at room temperature for 2 h. The MeOH was evaporated *in vacuo* and the pH was adjusted to 1-2 with HCl (1 M, aq). The aqueous suspension was extracted with CH_2_Cl_2_ (3x) and the organic layers were combined, dried (Na_2_SO_4_) and evaporated to yield the title compound as a light brown solid (313 mg, 0.99 mmol, 99%). ^1^H-NMR (500.23 Mhz, CDCl_3_) δ: 7.56 (td, ^4^J_H,F_ = 8.9 Hz (2x), ^3^J_H,H_ = 5.5 Hz, 1H), 6.99 (ddd, ^3^J_H,F_ = 9.1 Hz, ^3^J_H,H_ = 7.6 Hz, ^4^J_H,F_ = 1.9 Hz, 1H), 6.47 (s, 1H), 3.06 (m, 2H), 1.88 (sxt, ^3^J_H,H_ = 7.6 Hz, 2H), 1.06 (t, ^3^J_H,H_ = 7.4 Hz, 3H). ^13^C-NMR (125.78 MHz, CDCl_3_) δ: 158.4 (C_q_), 156.4 (C_q_), 123.3 (CH), 121.7 (C_q_), 112.2 (CH), 96.6 (C_q_), 54.2 (CH_2_), 17.2 (CH_2_), 12.9 (CH_3_). HR-MS (ESI, 4500V): m/z calculated for C_9_H_10_BrF_2_NO_2_S: 312.9584, found: (M+Na^+^): 337.9391.

### N-(2,4-difluoro-3-(tributylstannyl)phenyl)propane-1-sulfonamide (3)

A flame dried flask was charged with: *N*-(3-bromo-2,4-difluorophenyl)propane-1-sulfonamide (10, 500 mg, 1.59 mmol) and freshly distilled THF (15 mL). The solution was cooled to −80°C and to this was added to *N*-buthyllithium (2.19 ml, 3.50 mmol, 1.6M in hexanes). The obtained reaction mixture was stirred for 30 minutes, after which tributylchlorostannane (518 μL, 1.91 mmol) was added and the mixture was allowed to warm up to RT and stirred overnight. The mixture was quenched by the addition of water and subsequently extracted with CH_2_Cl_2_. Evaporation of the organic layer afforded the crude product, which was purified by flash column chromatography (Hexane/EtOAc, 9:1, v/v) to afford the title compound as a viscous oil (400 mg, 0.763 mmol, 48% yield). ^1^H-NMR (500.23 Mhz, CDCl_3_) δ: 7.53 (td, ^4^J_H,F_ = 9.1 (2x), ^3^J_H,H_ = 6.1 Hz, 1H), 6.85 (dd, ^3^J_H,F_ = 9.0 Hz, ^3^J_H,H_ = 5.7 Hz, 1H), 6.30 (s, 1H), 3.03 (m, 2H), 1.88 (sxt, ^3^J_H,H_ = 7.6 Hz, 2H), 1.55 (m, 6H), 1.35 (sxt, ^3^J_H,H_ = 7.3 Hz, 6H), 1.21 (m, 6H), 1.05 (t, ^3^J_H,H_ = 7.4 Hz, 3H), 0.91 (t, ^3^J_H,H_ = 7.3 Hz, 9H). ^13^C-NMR (125.78 Mhz, CDCl_3_) δ: 165.6 (C_q_), 163.7 (C_q_), 159.0 (C_q_), 125.9 (CH), 120.0 (C_q_), 111.5 (CH), 53.4 (CH_2_), 28.9 (3× CH_2_), 27.2 (3× CH_2_), 17.2 (CH_2_), 13.7 (3× CH_3_), 12.9 (CH_3_), 9.8 (3× CH_2_). HR-MS (ESI, 4500V): m/z calculated for C_21_H_37_F_2_NO_2_SSn: 525.1535, found: (M+Na^+^): 548.1502.

### Radiochemistry

#### Production of [^11^C]CO

[^11^C]CO_2_ was produced by the ^14^N(p,α)^11^C nuclear reaction performed in a 0.5% O_2_/N_2_ gas mixture (Linde gas, Schiedam, The Netherlands) using an IBA Cyclone 18/9 cyclotron (IBA, Louvain-la-Neuve, Belgium). Radioactivity levels were measured using a Veenstra (Joure, The Netherlands) VDC-405 dose calibrator. Radiosynthesis was performed using in-house built remotely controlled synthesis units [35]. After irradiation, [^11^C]CO_2_ was transferred to the experimental set-up and concentrated on a silica trap (50 mg silica gel, 100/80 mesh) at −196°C in liquid nitrogen. When the activity reached a maximum, the trap was heated and [^11^C]CO_2_ was passed over a gas purifier column (400 × 4 mm, silica gel, 100/80 mesh) using helium (40 mL.min^−1^) as a carrier gas. The purified [^11^C]CO_2_ was passed over a molybdenum reductor column (< 150 μm, 99.99%, Sigma Aldrich) at 850°C after which unreacted [^11^C]CO_2_ was trapped on an ascarite column and [^11^C]CO was collected on a silica trap (1 mg silica gel, 100/80 mesh) at −196°C. The transfer gas was switched from helium to xenon (Fluka ≥ 99.995, via Sigma Aldrich), [^11^C]CO was released by heating of the trap and transferred to the capped and closed reagent vial by a gentle xenon flow (2.0 mL.min^−1^). The reactions were performed after removal of the transfer needle and all experiments were carried out with the same vial type (1 mL, clear crimp vial, 10 × 32 mm, type 27333, Supelco, via Sigma Aldrich) and septum (11-mm aluminum crimp cap, 1.5-mm silicone/PTFE seal, Grace Alltech, Columbia, Maryland, USA) [29].

### [^11^C]Vemurafenib radiosynthesis

Pd_2_(dba)_3_ (2.4 mg, 10 μmol) was dissolved in dry THF (700 μL), and to this solution was added in sequence: triphenylarsine (6.5 mg, 20 μmol), 5-(4-chlorophenyl)-3-iodo-1H-pyrrolo[2,3-b]pyridine (2, 10.0 mg, 28.2 μmol) and *N*-(2,4-difluoro-3-(tributylstannyl)phenyl)propane-1-sulfonamide (3, 45.0 mg, 85.8 μmol). The vial was capped and connected to the transfer needle for [^11^C]CO. After transfer of [^11^C]CO to the reaction mixture (as described above) the vial was heated to 100°C for 5 min. followed by the introduction of a vent needle to the reaction vial and heating for an additional 2 min. at 130°C to evaporate the THF and remove excess gaseous [11]CO. The obtained residue was dissolved in semi-preparative HPLC eluent (3 mL, 65/35/0.1, MeCN/H_2_O/TFA, v/v/v) and subjected to purification by semi-preparative HPLC chromatography using an Grace Alltima C18 column (5 μ, 250 mm × 10 mm, Columbia, USA) using as eluent: 65/35/0.1, MeCN/H_2_O/TFA, v/v/v at a flow of 4 mL.min^−1^. Retention time of the product was 10.5 minutes. The collected fraction of the preparative HPLC purification containing [^11^C]vemurafenib was reformulated via solid phase extraction. The fraction was first diluted with 40 mL of water and the total mixture was passed over a preconditioned (10 mL of ethanol, 10 mL of water) tC18 plus Sep-Pak cartridge (Waters, Milford, Massachusetts, USA). The cartridge was then washed with 15 mL of sterile water and 0.5 mL of sterile 96% ethanol after which the product was eluted from the cartridge with 0.5 mL of sterile 96% ethanol. The ethanol was diluted to 10 volume percent with formulation solution (7.09 mM NaH_2_PO_4_ in 0.9% NaCl, w/v in water, pH 5.2) and the complete solution was filtered over a Millex-GV 0.22 μm filter into a sterile 20 mL capped vial. To provide a final solution of [^11^C]vemurafenib in 10% ethanol in saline (containing 7.09 mM NaH_2_PO_4_). Analysis of the product was performed by analytical HPLC. Retention time of the product was 6 min., radiochemical purity was > 99%. The specific activity was calculated against a calibration curve of vemurafenib using the analytical HPLC system and was found to be 55 ± 18 GBq/μmol.

### FACS analysis

For FACS analysis of BRAF expression of Colo829 and MeWo cells, the cells were first trypsinized and diluted with PBS to a final volume of 5 mL. Subsequently an aliquot of these cells (100 μL) were counted. The cells were centrifuged (1600 rpm, 4 min.; Hettich Universal 320, Hettich, Buford, Georgia, USA), the supernatant was removed and the pellet was taken up in fresh PBS (500 μL) and was centrifuged again (1600 rpm, 4 min., Eppendorf 5417R Microcentrifuge, Fisher Scientific, Waltman, Massachusetts, USA). The supernatant was removed and the pellet was taken up in icecold PBS (150 μL), cells were treated with paraformaldehyde (16% in water, Brunschwig Chemie, Amsterdam, The Netherlands) and incubated for 20 min. on ice for fixation, followed by washing with PBS (100 μL, twice). Subsequently cells were permeabilized with 0.1% sapponine (in PBS) at room temperature for 30 min. followed by centrifugation (1600 rpm, 4 min.). The cells were blocked by the addition of 0.1% sapponine /2% BSA (in PBS) for 30 min. at room temperature and centrifuged (1600 rpm, 4 min., Eppendorf 5417R Microcentrifuge). The BRAF antibody (purified mouse anti-BRAF, 250 μg//mL, 10 uL diluted with 90 uL 0.1% sapponine/2%BSA, BD Biosciences, San Jose, California, USA) was added and the cells were incubated for 30 min. at room temperature. Upon completion the cells were washed with 0.1% sapponine/2%BSA in PBS (100 μL) and the secondary antibody (Goat anti-mouse Ig FITC, 250 μg//mL, 4 uL in 96 uL 0.1% sapponine/2%BSA in PBS, BD Biosciences systems) was added and the cells were incubated in the dark for 30 min. at room temperature. Upon completion the cells were washed with PBS and taken up in fresh PBS for FACS analysis. Samples were measured by a two laser (488 nm blue laser and 635 nm red laser) Calibur flow cytometer (BD Biosciences) employing the 488 nm blue laser. Data was acquired and analyzed with Cell Quest software (BD Biosciences). Control experiments included treating the cells in the same manner, with exclusion of the primary antibody or the exclusion of both antibodies. The total signal was corrected by subtraction of the signal obtained with the negative control antibody from the total signal measured. Results are displayed as an average of three independent experiments (*N* = 3).

### Sequencing analysis of BRAF

For Colo829 and MeWO, the mutational status of *BRAF* exon 15 was assessed by HRM (high-resolution melting) assay followed by Sanger sequencing of HRM-PCR products with an aberrant melting curve, essentially as described previously [36].

### Cell titer blue assays

Vemurafenib stock solutions of 1 mM in DMSO were prepared and diluted with PBS to a concentration of 10 μM (1% DMSO). Cells (Colo829 or MeWo) were seeded in triplo in a 96 wells plate and treated with increasing concentrations of vemurafenib, from the above mentioned stock solution diluted with medium. After 120 h incubation at 37°C in a CO_2_ incubator, CellTiter-Blue (resazurin, Promega, Madesson, WI, USA) was added to the wells and the cells were incubated in the dark for 4 h at 37°C in a CO_2_ incubator after which the reaction was stopped by the addition of 3% SDS (50 μL) to each well. Finally the fluorescence was measured using a TriStar^2^ LB942 plate reader (Berthold Technologies, Bad Wildbad, Germany). Data was corrected for medium only and normalized to untreated cells (100% viability)

### Autoradiography on Colo829 and MeWo xenograft sections

Colo829 and MeWo xenograft sections (10 μm thickness) were pre-treated three times with 5 mM Tris-HCl buffer (pH 7.4) for 5 min.. Sections were dried under a gentle air flow before incubation for 30 min. with [^11^C]vemurafenib and the following conditions: (A) 5 mM Tris-HCl, pH 7.4; (B) 5 mM Tris-HCl, pH 7.4 and isotopically unmodified vemurafenib at 100 μM (*n* = 4 for each incubation condition). Washing was performed using Tris-HCl (3×) followed by dipping in ice cold water. After drying in an air stream, tumor sections were exposed to a phosphor-imaging screen for 15 min. Data was quantified as average signal intensity per surface area and were represented relative to the tracer only (A) conditions, which was set at 100%. Error bars indicate standard deviations. Quantification of binding was performed using ImageQuantTL v8.1.0.0 (GE Healthcare, Buckinghamshire, UK) by drawing regions of interest around the full tumor sections.

### *In vitro* cell uptake experiments

Colo829 or MeWo cells (1*10^6) suspended in growth medium were incubated in triplicate in centrifuge tubes at 37°C with 1 MBq of [^11^C]vemurafenib (corresponding to 0.02 nmol of vemurafenib) for 30 min. Upon completion of the incubation period the tubes were centrifuged (5000 rpm, 5 min, 0°C, Hettich universal 16, Depex B.V., the Netherlands) and the supernatant was removed and collected. Subsequently the cells were gently resuspended in PBS and centrifuged again. This procedure was repeated twice and PBS wash steps were collected. All fractions (cells, supernatant and PBS) were counted for radioactivity using a gamma counter (Wallac 1210 Compugamma, PerkinElmer, Waltham, MA, USA) and the cell bound/internalized percentage of [^11^C]vemurafenib was determined as the fraction of activity in the cell pellet divided by the total activity (cells, supernatant and PBS) corrected for background (medium only) and vehicle treated conditions.

### Plasma metabolite analysis

Athymic nu/nu mice were injected with 20-25 MBq of [^11^C]vemurafenib (corresponding to 0.36-0.45 nmol of vemurafenib), in the ocular plexus under isoflurane anesthesia (2% in 1 L·min^−1^). The mice were sacrificed at 15 (*n* = 3) and 45 (*n* = 3) min. p.i.. At these time points, about 1.0–1.5 mL of blood was collected via a heart puncture from each mouse. Blood was collected in a heparin tube and centrifuged for 5 min. at 4000 r.p.m. (Hettich universal 16, Depex B.V., The Netherlands). Plasma was separated from blood cells and about 1 mL of plasma was diluted with 2 mL of 0.1 M hydrochloric acid and loaded onto a tC18 Sep-Pak cartridge, which was pre-activated by elution with 3 mL of MeOH and 6 mL of water, respectively. The cartridge was washed with 5 mL of H_2_O to collect polar radioactive metabolites. Thereafter, the tC18 Sep-Pak cartridge (Waters, Milford, Massachusetts, USA) was eluted with 2 mL of MeOH and 1 mL of H_2_O to collect the mixture of non-polar metabolites. The mixture of non-polar metabolites was analyzed using HPLC to determine the percentage of intact [^11^C]vemurafenib. HPLC was performed on a Dionex (Sunnyville, California, USA) Ultimate 3000 system, equipped with a 1 mL loop. As a stationary phase a Phenomenex (Torrance, California, USA) Gemini C18, 250 × 10 mm, 5 μm was used. The mobile phase was a gradient of A = acetonitrile and B = 0.1% TFA in H_2_O. The HPLC gradient ran for 12.0 min. decreasing the concentration of eluent B from 60% to 20% in 10 min. followed by 2 min. of 20% eluent B at a flow rate of 3.5 mL · min^−1^. Recovery of radioactivity was > 95%.

### Biodistribution studies

Nude mice (Athymic nu/nu, Harlan, Horst, The Netherlands) bearing two tumors (obtained by injection of MeWo or Colo829 cells, 2*10^6 per site) of the same xenograft line on their left and right flank, received an injection of 25 MBq [^11^C]vemurafenib (corresponding to 0.45 nmol of vemurafenib) via the ocular plexus. The mice were sacrificed and dissected at 60 min. post-injection. Blood, urine, skin, left tumor, right tumor, muscle, heart, colon, lung, liver, kidney and brain were collected, weighed and counted for radioactivity in a gamma counter (Wallac 1210 Compugamma, PerkinElmer, Waltham, MA, USA). Biodistribution data are expressed as percentage of injected dose per gram (%ID/g) of tissue for each organ.

### Statistical analysis

Statistical analysis was performed using graphpad PRISM (v 5.02, Graphpad Software Inc). Cell binding, autoradiography and tumor uptake in biodistribution studies were compared between cell lines using a two-tailed Student's *t-test* for paired data (a statistically significant difference was defined as a *P-value* of < 0.05).
